# Bolt gun injury to central forehead, sagittal sinus and frontal lobes: A case report

**DOI:** 10.1016/j.ijscr.2025.111559

**Published:** 2025-06-22

**Authors:** Ning Zhu, Rondhir Jithoo, Jeffrey V. Rosenfeld

**Affiliations:** a Department of Neurosurgery, The Alfred Hospital, Melbourne, Australia; b Department of Surgery, Monash University, Melbourne, Australia

**Keywords:** Bolt gun injury, Penetrating brain injury, Traumatic brain injury, Bifrontal craniectomy

## Abstract

**Introduction and importance:**

Bolt gun injuries to the head are rare and are usually in the context of suicide. Bolt guns are used in the meat processing industry to induce unconsciousness prior to slaughter. A bolt penetrates 7–10 cm through the skull into the brain and then is retracted by a spring. Mortality has been reported to be up to 80–90 %, with survivors left with severe neurological and functional deficits.

**Case presentation:**

A 24-year-old man presented with a self-inflicted bolt gun injury to the central forehead. He presented confused and agitated to a rural emergency department and was subsequently intubated and transferred to a tertiary trauma centre. CT demonstrated a midline frontal bony defect with an underlying 8.5 cm wound tract into both frontal lobes. There was associated intracranial haemorrhage and impacted skin and bone fragments within the wound tract. CTA/V showed an injury to the superior sagittal sinus but intact arterial vessels. He underwent a bifrontal craniectomy, débridement and evacuation of haematoma with ICP monitor placement. The patient demonstrated a remarkable recovery at 3 months with only mild cognitive impairment.

**Clinical discussion:**

CT imaging including angiography assesses extent of parenchymal and vascular injury. Decompression of the brain and careful debridement off the brain reduced the risk of infection but prevented iatrogenic injury. Prophylactic treatment with broad-spectrum antibiotics prevented intracranial infection.

**Conclusion:**

This case report highlights multiple lessons which contributed to successful management of a bolt gun injury to the head: preoperative CT angiography, craniectomy if indicated, cautious debridement and prophylactic antibiotics.

## Highlights

• CT/A assesses parenchymal and vascular injury
• Craniectomy prevents cerebral perfusion compromise from brain swelling.
• Meticulous but cautious débridement prevents infection and iatrogenic injury
• Prophylactic antibiotics prevent meningitis/cerebritis

## 1 Introduction

Bolt gun injuries to the head are rare, with most neurosurgeons likely to never see such an injury [1]. Few reports available in the literature [2], which have mostly originated from central European countries [3].

Bolt guns are used in the meat processing industry to induce unconsciousness prior to slaughter [4]. A bolt penetrates 7–10 cm through the skull into the brain and then is retracted by a spring [2]. This mechanism leads to specific injuries: a circular skin and bone defect, a long-wound canal with skin, other skin elements and bone fragments at its end and the absence of an exit wound [5].

Bolt gun injury to the head in humans is predominantly suicidal events [3]. Extensive destruction of brain structures including vascular injury and delayed intracranial infection with mixed bacterial growth may occur [6]. Subsequently mortality has been reported to be up to 80–90 %, with survivors left with severe neurological and functional deficits [1,4]. This case report is compliant with SCARE criteria [7].

## 2 Case presentation

A 24-year-old man was referred from a rural hospital to our tertiary specialist neurosurgery unit following a self-inflicted penetrating brain injury to his central forehead. He was found confused and with a bleeding forehead wound in a nearby park after a suicide letter to his sister was discovered. His relatives uncovered a bolt gun near where he was found that had been obtained from his workplace at a local piggery. On arrival to the rural hospital by ambulance, he was agitated with a Glasgow Coma Scale (GCS) of 13. He was subsequently intubated to facilitate a CT scan. He had a past medical history of depression and was not on any medications (including anticoagulation). On arrival to our hospital, the patient was heavily sedated and intubated, with 2.5 mm reactive pupils bilaterally.

## 3 Investigations

**Fig. 1:**
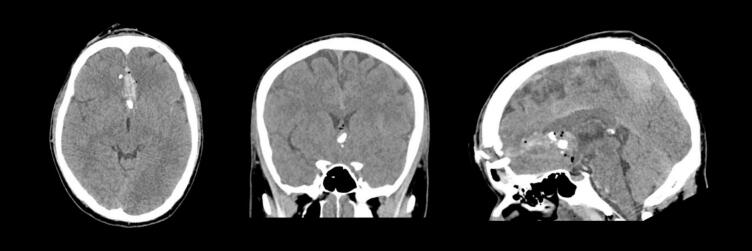
Preoperative non contrast CT Brain (Axial/Coronal/Sagittal views).

The patient's initial CT at the rural hospital showed a comminuted fracture of the frontal bone involving both frontal sinuses, with displaced bone fragments extending into the frontal lobes. The wound tract measured 8.5 cm, with debris and locules of gas along the tract. There was associated intraparenchymal, subdural and subarachnoid haemorrhage as well as oedema and swelling of the bilateral frontal lobes (see Fig. 1, Fig. 4A).

**Fig. 2:**
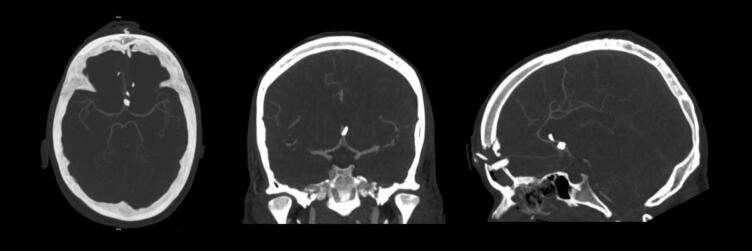
Preoperative CT brain angiogram (Axial/Coronal/Sagittal views).

On arrival to our tertiary specialist centre, we performed a repeat CT Brain including an angiogram. The CT brain was unchanged. The CT angiogram showed multiple osseous fragments adjacent to the bilateral A2 anterior cerebral arteries (ACA) without evidence of arterial injury (see Fig. 2).

## 4 Treatment

The patient was sedated and intubated prior to transfer. Intravenous Cefazolin 2 g and Levetiracetam 2 g was given. On arrival, intravenous Cefepime 2 g, Vancomycin 2 g, Metronidazole 500 mg and tranexamic acid 1 g were administered.

The patient received a bifrontal craniectomy, wound débridement, evacuation of haematoma, removal of accessible bone fragments, cranialisation of frontal sinus and the insertion of an ICP monitor.

**Fig. 3:**
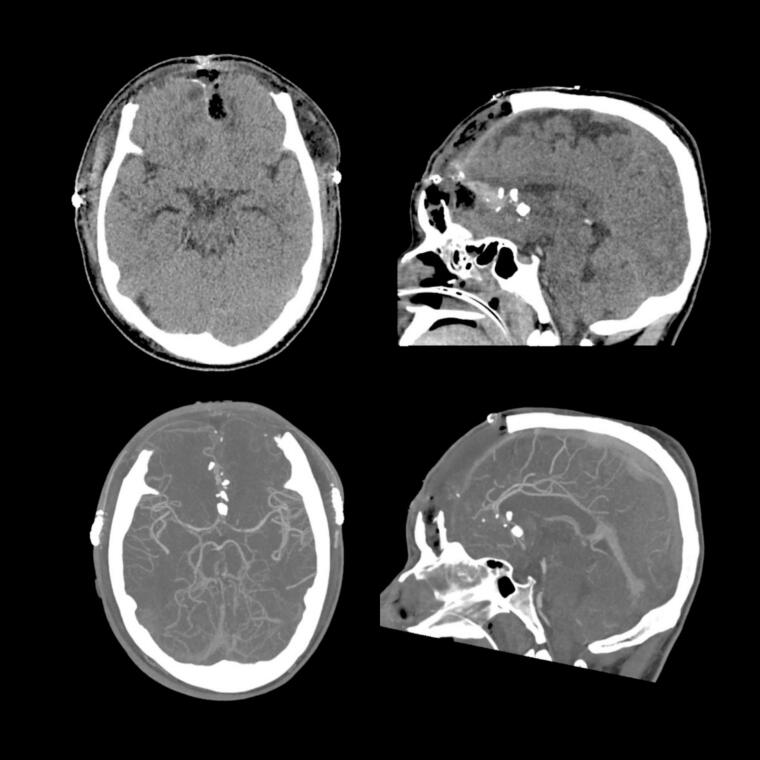
Post operative CT Brain and CT Brain Angiogram (Axial/Sagittal views).

**Fig. 4:**
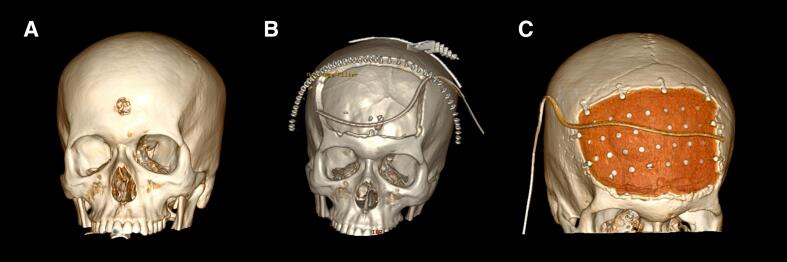
3D reconstructions of preoperative CT Brain (A), post decompressive bifrontal craniectomy CT Brain (B), post cranioplasty CT Brain (C).

There was a complete hair shave and preliminary scrub of scalp with cetrimide and alcohol solution to remove any debris and blood. A bi-coronal incision and a generous bifrontal craniotomy were performed (see Fig. 3, Fig. 4B). U-shaped durotomies were performed bilaterally. Significantly swollen brain was identified with cortical lacerations and contusions of the frontal lobe bilaterally. The dura was subsequently left open. The sagittal sinus injury required ligation. A decision to leave the bone off (craniectomy) was made due to the identified swelling of the brain and anticipated progression of swelling secondary to brain oedema, sinus injury and intracranial haemorrhage. The bone flap was discarded, instead of stored, due to risk of infection. Subdural and intraparenchymal haemorrhage was evacuated. The brain was meticulously but cautiously debrided. Superficial bone fragments were performed, while deeper fragments were intentionally left in situ to minimise risk of iatrogenic injury. Care was taken to visualise and protect the intact ACAs. The frontal sinuses were cranialized by drilling out the mucosa and plugging of ostia with muscle grafts from the left temporalis, gel foam and Tisseal glue. Synthetic dural substitutes were laid on top of the open dura and covered with Tisseal and Surgicel. The entry wound was closed internally but left to heal externally by secondary intention.

Post operatively, the patient was kept sedated in the ICU for management of intracranial pressure and slowly weaned off sedation. Tranexamic acid 1 g was given 8 hourly for a further 24 h. The Infectious Disease team was consulted and intravenous Cefepime, Vancomycin and Metronidazole were given for 7 days and ceased. Levetiracetam was administered at 500 mg twice a day for 7 days. There was no post operative CSF leak.

## 5 Outcomes

The patient demonstrated a remarkable recovery in the short and long term. Day 1 after injury and surgery, he was noted to be opening his eyes to voice and obeying commands with an ICP of 6-9 mmHg. He was extubated on day 6. Inpatient psychiatry assessment diagnosed untreated major depressive disorder and subsequently commenced Sertraline. He was transferred to a dedicated brain injury rehabilitation facility on day 24.

**Fig. 5:**
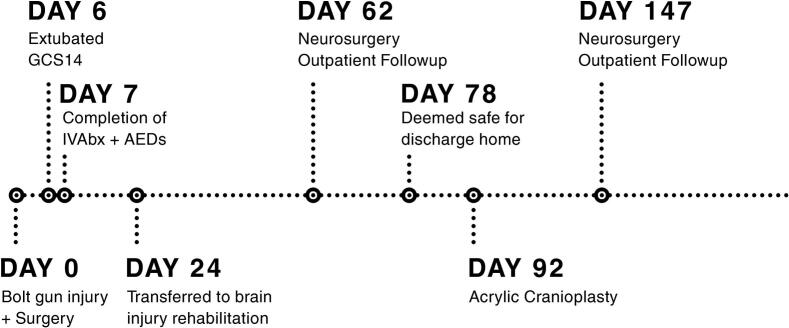
Timeline of patient's journey.

Patient was treated with a coordinated multidisciplinary effort by occupational therapy, physiotherapy, psychology, neuropsychology, psychiatry, speech pathology and social work. Focuses were on adequate titration of antidepressant medication, involvement of family members in patient's daily rehabilitation program, a neurodevelopmental, family centred and future focused approach, formulation of a consistent daily routine, use of external memory aids, developing patient's mental health literacy and awareness into mental health challenges and creating a management plan to support the patient in the community. Assessment by a neuropsychologist at 2 months advised significant gains in cognitive recovery, with good general intellectual abilities (including abstract visual and verbal reasoning), relative strength in visual problem solving and capacity to learn from mistakes. He had mild-moderate deficits in high level attentional functioning and mild to moderate memory impairment. He was deemed safe for discharge home at 2.5 months. He was transferred from rehabilitation back to our specialist tertiary hospital at 3 months and underwent acrylic cranioplasty with good cosmetic result (see Fig. 4C). He was discharged home 2 days post cranioplasty and continued cognitive and functional rehabilitation in the community. Psychiatry assessment at 3 months showed marked improvements in mood, engagement, and warmth, with optimism about the future. Patient was followed up in the Neurosurgery Outpatient Clinic 6 weeks post cranioplasty (5 months post injury) (see Fig. 5).

## 6 Discussion

Our case describes management of a bolt gun injury to the head without significant complication or severe long term functional impairment. These injuries usually occur in the context of suicide and carry a high rate of morbidity, up to 80–90 % mortality [2]. Skin defects are small, and no projectiles are found in the wound tract [4,8]; however, there is extensive parenchymal injury, damage to large blood vessels and impacted pieces of skin and skin elements in the wound canal [6]. By contrast, in nail gun injuries to the head, the nails remain in the brain.

Our patient sustained a central forehead entry point. With a wound tract depth of 8.5 cm, the bolt penetrated deep enough to contact the anterior cerebral arteries but was fortunate to have passed just to the left side of these vessels. This likely contributed to the patient's good outcome. Gnjidic et al. found that six of out seven successful suicides from bolt gun injuries were due to injury to large arterial vessels [1].

CT imaging including CT angiography allows for instant assessment of the extent of parenchymal injury, intracerebral haemorrhage, midline shift and vascular injury (including the major venous sinuses) [9]. In our case, this identification of the superior sagittal sinus injury, the intact anterior cerebral arteries and the extent of intracerebral injury assisted with surgical planning.

Pesak et al. reviewed 11 cases of bolt gun injury from 2016 to 2023: 5 died, 5 had severe disability and 1 made a good recovery. A significant finding was that many patients developed diffuse brain oedema with compromised brain perfusion [8]. In penetrating brain injuries, a projectile (like the bolt of a bolt gun) exerts a high pressure on a small contact area. This can create secondary projectiles from bone fragments, form a permanent cavity after crushing of the soft tissues and cause powerful expansion of a temporary cavity [10]. Animal models have demonstrated that the significant role of kinetic energy transfer of the bolt in causing both focal and diffuse brain damage. There is direct physical injury at the penetration site, in the wound path created by the bolt and in paths of secondary missiles (bone, skin fragments). The bolt imparts high radial forces to surrounding tissue, with shock waves, coup and contre coup forces, shear forces and cerebral haemorrhage causing diffuse haemorrhage and ruptured capillaries at some distance from the wound path [11,12].

Recent literature has advocated for more aggressive management for penetrating and blast injury patients, including decompression of the brain [9,13]. Joseph et al. found that the rate of survival increased from 10 % to 46 % with aggressive management [14]. In a military setting, early cranial decompression has been shown to increase survival of penetrating brain injury [15]. We performed a bifrontal craniectomy given the brain swelling observed intraoperatively and the high likelihood of further progression given the pathophysiology of penetrating brain injury and the ligated superior sagittal sinus as well as observed and radiological intracranial haemorrhage, contused brain and brain oedema.

Newer evidence has advocated for less aggressive débridement and retrieval of deep bone fragment to avoid iatrogenic injury to deep vessels and other structures [13]. This contrasts with previous aims to completely removal of all bone fragments, foreign bodies and all devitalised tissue to reduce the risk of infection [1,16]. We performed a meticulous débridement but did not attempt to retrieve some deep bone fragments adjacent to the ACAs. Like Pesak et al., we cranialized the frontal sinus [8]. The superior sagittal sinus injury in our patient was in the anterior third, which we were able to ligate without significant problems [17].

Bolt gun injuries often develop purulent meningitis and cerebritis due to these guns being heavily contaminated and the impacted skin and debris within the wound canal [1]. Recent studies have demonstrated lower morbidity and mortality with prophylactic antibiotic treatment in penetrating head injuries [18,19]. Various guidelines including that of the American Association for the Surgery of Trauma Critical Care Committee, have recommended a short course of prophylactic broad-spectrum antibiotics for penetrating brain injury [9,20,21]. The United States Department of Defence Center for Excellence for Trauma Guidelines advise Cefazolin 2 g Q8H or Clindamycin 600 mg Q8H for an unspecified duration, with the addition of Metronidazole 500 mg Q8-12H for visibly contaminated wounds. The United States Army Center for Surgical Research recommends Cefazolin 1 g Q8H for 5 days and extension of this duration if there is gross contamination of the wound [18]. Our local Infectious Diseases specialists were consulted and our patient was given antibiotics on admission, intraoperatively and post-surgery for 7 days. This included antibiotics normally associated with meningitis and cerebritis (Cefepime, Vancomycin and Metronidazole). Our patient was able to avoid an intracranial infection.

CRASH-3 demonstrated that tranexamic acid within 3 h reduced head injury related death without adverse effect or complication [22]. Our patient was given tranexamic acid within 3 h of head injury and 8 hourly for 24 h post injury.

Prophylactic anti-epileptic drugs are generally recommended for the first week after penetrating brain injury [23]. Our patient was given 7 days of Levetiracetam and has not had a seizure.

A strength of this case was the adherence of management to the most up to date literature and the associated favourable outcome for the patient. There were no significant limitations of the case.

## 7 Conclusion

Bolt gun injuries to the head carry a high rate of morbidity and mortality. Our patient demonstrated a remarkable recovery at 3 months with only mild cognitive impairment. CT with angiography enabled immediate assessment of the extent of parenchymal injury and the exclusion of vascular injury. Bifrontal craniectomy accommodated brain swelling and prevented cerebral perfusion compromise. Meticulous but cautious débridement reduced the risk of infection and prevented iatrogenic injury to deep vessels and other structures. Prophylactic broad spectrum IV antibiotic coverage prevented development of meningitis. We believe these were pertinent steps which enabled successful management of a bolt gun injury to the head and can be applied to future cases to increase the likelihood of a favourable outcome.

## Consent

Written informed consent was obtained from the patient for publication of this case report and accompanying images. A copy of the written consent is available for review by the Editor-in-Chief of this journal on request.

## Ethical approval

Case reports are exempt from needing ethical approval at our institution.

## Guarantor

NZ is the Guarantor for this study and accepts full responsibility for the work and the conduct of the study, had access to the data and controlled the decision to publish. RJ and JR provided written permission for the current version of the manuscript to be published.

## Research registration number

This study is not ‘First in Man’ and as such was not registered.

## Funding

This research did not receive any specific grant from funding agencies in the public, commercial, or not-for-profit sectors.

## Author contribution

NZ – Data curation, formal analysis, investigation, methodology, writing, review and editing RJ – Supervision, conceptualization, review and editing JR – Supervision, conceptualization, review and editing

## Conflict of interest statement

The authors declare that they have no known competing financial interests or personal relationships that could have appeared to influence the work reported in this paper.

## Glossary

CT: Computed tomography
CTA/V: Computed tomography angiogram/venogram
AED: antiepileptic drugs
ACA: anterior cerebral arteries
GCS: Glasgow Coma Scale
ICP: intracranial pressure
IV: intravenous
ICU: intensive care unit
